# Repurposing Treatment of Wernicke–Korsakoff Syndrome for Th-17 Cell Immune Storm Syndrome and Neurological Symptoms in COVID-19: Thiamine Efficacy and Safety, In-Vitro Evidence and Pharmacokinetic Profile

**DOI:** 10.3389/fphar.2020.598128

**Published:** 2021-03-02

**Authors:** Vatsalya Vatsalya, Fengyuan Li, Jane Frimodig, Khushboo S. Gala, Shweta Srivastava, Maiying Kong, Vijay A. Ramchandani, Wenke Feng, Xiang Zhang, Craig J. McClain

**Affiliations:** ^1^ Department of Medicine, University of Louisville, Louisville, KY, United States; ^2^ Robley Rex VA Medical Center, Louisville, KY, United States; ^3^ University of Louisville Alcohol Research Center, Louisville, KY, United States; ^4^ Envirome Institute, University of Louisville, Louisville, KY, United States; ^5^ Department of Bioinformatics and Biostatistics, University of Louisville, Louisville, KY, United States; ^6^ National Institute on Alcohol Abuse and Alcoholism, NIH, Bethesda, MD, United States; ^7^ Department of Pharmacology and Toxicology, University of Louisville, Louisville, KY, United States; ^8^ University of Louisville Hepatobiology and Toxicology COBRE, Louisville, KY, United States; ^9^ Department of Chemistry, University of Louisville, Louisville, KY, United States; ^10^ Center for Regulatory and Environmental Analytical Metabolomics, University of Louisville, Louisville, KY, United States

**Keywords:** cytokine storm, COVID-19, IL-17, pandemic, thiamine

## Abstract

Coronavirus disease identified in 2019 (COVID-19) can be complicated by the Th17 cell-mediated IL-17 proinflammatory response. We tested if thiamine can effectively lower the Th17 response in a clinical study [Proinflammatory state in alcohol use disorder patients termed as disease controls (DC)] and corroborated the results using an *in vitro* study. We developed an effective dose range and model for key pharmacokinetic measures with the potential of targeting the cytokine storm and neurological symptoms of COVID-19. Three-week 200 mg dose of thiamine was administered to sixteen DC patients. Eight healthy volunteers (HV) were also included in this investigation. A subsequent *in vitro* study was performed to validate the effectiveness of thiamine [100 mg/day equivalent (0.01 μg/ml)] treatment in lowering the Th17 proinflammatory response in a mouse macrophage cell line (RAW264.7) treated with ethanol. Based on recent publications, we compared the results of the IL-17 response from our clinical and *in vitro* study to those found in other proinflammatory disease conditions (metabolic conditions, septic shock, viral infections and COVID-19) and effective and safe dose ranges of thiamine. We developed a pharmacokinetic profile for thiamine dose range as a novel intervention strategy in COVID-19. DC group showed significantly elevated proinflammatory cytokines compared to HV. Thiamine-treated DC patients showed significant lowering in IL-17 and increase in the IL-22 levels. In humans, a range of 79–474 mg daily of thiamine was estimated to be effective and safe as an intervention for the COVID-19 cytokine storm. A literature review showed that several neurological symptoms of COVID-19 (∼45.5% of the severe cases) occur in other viral infections and neuroinflammatory states that may also respond to thiamine treatment. Thiamine, a very safe drug even at very high doses, could be repurposed for treating the Th17 mediated IL-17 immune storm, and the subsequent neurological symptoms observed in COVID-19. Further studies using thiamine as an intervention/prevention strategy in COVID-19 patients could identify its precise anti-inflammatory role.

## Introduction

Viral diseases and wide-spread outbreaks have adverse health-related consequences worldwide. Emerging infectious diseases (EID) include viral pathogens that have shown higher incidence of human infection in the past several decades and raise concerns regarding increased ongoing/future prevalence (Hassell et al., 2017). Coronavirus is recognized as an EID that has become a challenging and aggressive infection with high morbidity and mortality in humans (Poon and Peiris, 2020). SARS-CoV-2 [severe acute respiratory syndrome coronavirus 2; causes coronavirus disease (COVID-19)] was identified in 2019, has become a pandemic, and is a priority healthcare concern in the year 2020 (HuiDS et al., 2020).

In viral infections, tissue inflammation is driven by multiple proinflammatory and immunoregulatory signals (Glass et al., 2003; Tay et al., 2020). The pathological progression of COVID-19 has multiple clinical stages and may present with the cytokine storm syndrome (Pedersen and Ho, 2020) and immunosuppression (Mehta et al., 2020). Interleukin-17 (IL-17) is a cytokine (Aggarwal and Gurney, 2002) that is often involved in a proinflammatory response in the cytokine storm of viral infections in humans (Yuan et al., 2010; Jain et al., 2013; Reed et al., 2015) and experimental mice model (Zhang et al., 2009). It can also promote respiratory viral infections (Mukherjee et al., 2011), tissue pathology (Klatt et al., 2012; Li et al., 2012; Du et al., 2013), and neurological manifestations (Ye et al., 2020). Th17 cells also produce Interleukin-22 (IL-22), which plays a protective/anti-inflammatory role, and it is dysregulated in several proinflammatory conditions (Eyerich et al., 2017). Thus, a therapy that could alleviate the Th17 mediated pro-inflammatory response (Pacha et al., 2020) might be effective in attenuating the cytokine storm observed in COVID-19 patients.

Thiamine, a vitamin and dietary supplement (Cooper and Pincus, 1979), has anti-oxidant properties (Cooper and Pincus, 1979; Thornalley, 2005). High levels of cytokines (for example, IL-1β and IL-6) may occur in thiamine deficient subjects and can be associated with oxidative stress and inflammation (Neri et al., 2011; de Andrade et al., 2014). Importantly, thiamine administration could inhibit production of these cytokines, increase anti-inflammatory activity (Shahmiri et al., 2013; Menezes et al., 2017), and potentially alleviate neuroinflammatory symptoms of viral origin (Protheroe and Mellor, 1991; Brechtelsbauer et al., 1997).

We tested the efficacy of a three-week thiamine treatment in modulating the Th17 proinflammatory response in a human disease control model of conditions associated with inflammation. To validate the effectiveness of thiamine in treating the proinflammatory response from the human study, we conducted an *in vitro* experiment to test the effects of thiamine treatment in alleviating ethanol mediated immune dysregulation in a mouse macrophage cell line, RAW264.7. We investigated the Th17 cells proinflammatory cytokine response (namely IL-17) in both healthy controls and individuals with high inflammatory response. This was done to estimate the effects of various doses of thiamine that have shown efficacy in alleviating the Th17 associated cytokine response. We assessed the pharmacokinetics of the oral thiamine dosing. Lastly, we also examined the neurological symptoms of COVID-19 that could possibly be treated with thiamine.

## Materials and Methods

### Study Participants

This investigation was approved under two large clinical investigations that were conducted at the University of Louisville (NCT#01809132, HV cohort), and the National Institute on Alcohol Abuse and Alcoholism (NIAAA) (NCT#00106106, DC cohort) at the National Institutes of Health (NIH), Bethesda MD. The studies were approved by the NIH Institutional Review Board (IRB) committee and the UofL IRB (IRB # 12.0427). Sixteen age- and sex-matched male and female alcohol use disorder (AUD) patients [Termed as disease controls (DC) in this investigation] between 21 and 65 years of age with both present and past heavy drinking profile participated as the DC for thiamine administration. All study patients were diagnosed with AUD based on DSM-IV TR criteria. All study patients received daily doses of open label thiamine (100 mg twice daily = 200 mg per day) (Thomson, 2000) for 3-weeks after completion of the consenting process. All patients also received standard clinical inpatient care as part of the medical management for their AUD, including counseling. Detailed information on subject recruitment and management can be obtained from several of our previous publications (Vatsalya et al., 2016; Vatsalya et al., 2018; Vatsalya et al., 2019; Vatsalya et al., 2020). We also included eight healthy controls in this study for comparison with DC. Demographic data were collected from all the participants. Baseline (HV and DC) and post-treatment (DC only) blood (after the completion of 3-weeks of thiamine dosing) were collected, processed (for plasma extraction), frozen at −80°C. They were subsequently thawed and assayed.

### Laboratory Assays and Therapeutic Model on Th17 Inflammation Axis

1. Cytokine assays

Plasma levels of proinflammatory cytokines, IL-1β, IL-6, and IL-10 were obtained by multianalyte chemiluminescent detection using Multiplex kits (Millipore, Billerica, MA) on the Luminex platform (Luminex, Austin, TX), according to manufacturers’ instructions.

(2) Analysis of IL-17 and IL-22 in a set of AUD patients for designing proof-of-concept experimental model

We performed analyses for IL-17 and IL-22 on human plasma samples to estimate the Th17 inflammatory response, with the goal of developing an *in vitro* mechanistic experimental model to test the efficacy of thiamine. The plasma levels of IL-17 and IL-22 in eight healthy volunteers were also included in this study for comparison. IL-17 and IL-22 were detected in plasma using Human IL-17A (now called IL-17) High Sensitivity ELISA Kits (BMS2017HS, Invitrogen) and Human IL-22 ELISA Kits (BMS2047, Invitrogen) per the manufacturer’s instructions. Results were read on a Spectra Max Plus 384 plate reader and modeled using their SoftMax Pro software (Molecular Devices, San Jose, CA).

(3) Cell culture

RAW 264.7 cells (mouse macrophage cell line) were cultured in Dulbecco's modified Eagle's medium (DMEM, Invitrogen), supplemented with 10% fetal bovine serum (FBS) and 1% penicillin/streptomycin. Cells were seeded in a 24-well culture plate and maintained at 37°C in a 5% CO_2_ incubator for 3 days. The 0.02 μg/ml treatment dose was equivalent to the 200 mg/day thiamine dose [approximate blood AUC = 204 nmol/L (Smithline et al., 2012)] given to the patients. Cells were then treated with thiamine (Vit B1 [V_B1_] as shown in Supplementary Figure S1) at a concentration of (0.01 μg/ml) for 2 h (in a preventive paradigm), followed by 80 mM ethanol treatment for 22 h, for a total of 24 h of treatment to determine the minimum effective level of thiamine to reduce the Th17 response. Cells were then washed with PBS and collected with Trizol reagent for the isolation of RNA. RNA samples were reverse transcribed to cDNA and used for qRT PCR analysis of cytokine expression (IL-17, IL-22). Cell viability was not affected by thiamine or EtOH treatment at the doses used in the experiments.

(4) RNA isolation and real-time RT-PCR

**FIGURE 1:**
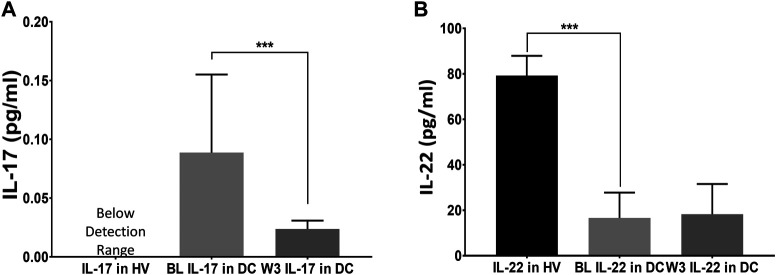
Efficacy of thiamine treatment on Th17 cell derived response for IL-17, and IL-22 cytokines. Levels of IL-17 and IL-22 in healthy volunteers (HV) at baseline; and Disease Controls [DC or (alcohol use disorder, AUD)] patients exhibiting a proinflammatory response (*n* = 16) at baseline; and anti-inflammatory normalization of cytokines tested after the completion of three-week (W3) thiamine treatment. A drop in IL-17 **(A)** coupled with a mild increase in IL-22 **(B)** at W3 was observed compared to the baseline levels. BL: baseline, W3: three-week of thiamine treatment. Data are presented as M ± SD. Statistical significance was set as *p* < 0.05.

Total RNA was extracted from the cells using Trizol reagent (500 µL/well) according to manufacturer’s instruction (Life Technologies, Carlsbad, CA) and reverse-transcribed using cDNA Supermix (QuantaBio, Beverly, MA). Quantitative real-time PCR was performed on an ABI 7500 real-time PCR thermocycler and SYBR green PCR Master Mix (Applied Biosystems, Foster City, CA) was used for quantitative real-time PCR analysis. The relative quantities of target transcripts were calculated from duplicate samples after normalization of the data against the housekeeping gene, mouse 18S. Relative mRNA expression was calculated using comparative Ct method. Test was conducted thrice (training, test and validation steps) and the results from the second tests were used. The following primer pairs were used:

Results are available in the supplement section.

### Development of the Pharmacokinetic Model for Dose Titration of Thiamine

We used dosing guidelines for thiamine as mentioned at the Medline Plus (https://medlineplus.gov/druginfo/natural/965.html#Safety, last reviewed as of August 5, 2020), and from peer reviewed publications from PubMed (https://pubmed.ncbi.nlm.nih.gov/; [searched and collected until August 5, 2020]). We used available dosing guidelines from Medline Plus for healthy individuals both for dietary supplementation and vitamin deficiency status. We also reviewed and incorporated thiamine dose levels (lower and higher range) from other disease conditions; namely metabolic conditions (Mandel et al., 1984; Riaz et al., 2011), septic shock (Marik et al., 2017; Moskowitz et al., 2017), viral diseases (Mouly, 1996; Arici et al., 2001; Margolis et al., 2014) and Leigh’s disease (Di Rocco et al., 2000) (Medline Plus: Thiamine). We also included the recorded thiamine dose levels from the DC group (AUD with Wernicke Korsakoff Syndrome, WKS (Day et al., 2013); from our clinical study) as one of the pro-inflammatory conditions.

**FIGURE 2:**
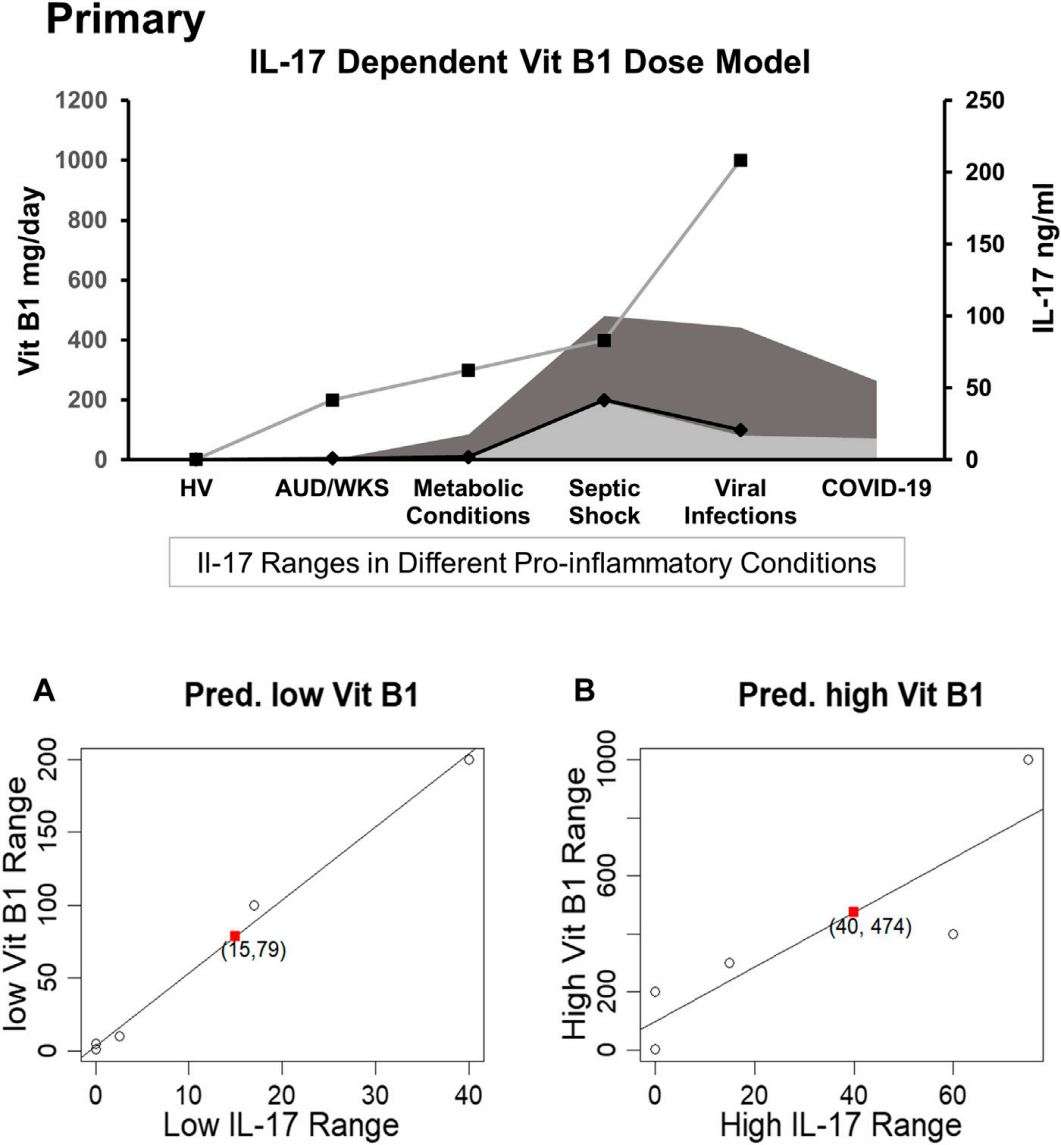
PRIMARY: A dose titration by disease and proinflammatory Th17 status model of thiamine administration with parallel representation of Th17 proinflammatory response in various groups including healthy volunteers and disease groups with pro-inflammatory response. **(A)** Linear regression model is predictive for the relation between the low dose thiamine (Vit B1) range vs. low IL-17 ranges based on known observed pairs from different patients; lower range of vit B1 dose (79 mg/daily) corresponding to lower range of IL-17 (15 ng/ml) in the COVID-19 patients. Dark grey shade depicts higher IL-17 response and lighter grey shade shows lower IL-17 response in various proinflammatory conditions. **(B)** Linear regression shows the predictive model for the relation between a higher range of Vit B1 dose vs. a higher range of IL-17 levels, derived from the known observed pairs from different patients; higher range of Vit B1 dose (474 mg/daily) corresponding to higher range of IL-17 (40 ng/ml) reported in COVID-19 patients. High Range Thiamine Dose: Left *Y*-axis (primary). Low Range Thiamine Dose, Low Range IL-17 levels, and High range IL-17 levels: Right *Y*-axis (Secondary).

We compared the reference range of levels of the Th17 cytokine (IL-17) response in disease/health conditions in humans as published in the recent findings concerning COVID-19’s cytokine storm data (Liu et al., 2020a; Huang et al., 2020; Wu and Yang, 2020). A Th17 proinflammatory response for the potential range of IL-17 levels was also developed for healthy volunteers (HV, from our study cohort), metabolic conditions (Kotake et al., 1999; Pirowska et al., 2018), DC (or alcohol use disorder patients from our study cohort), septic shock (Brunialti et al., 2012; Li et al., 2015), and viral infections (Crowe et al., 2009; Mukherjee et al., 2011). IL-17 data on severe COVID-19 patients (as mentioned above) were collected from the recently peer-reviewed published articles found in PubMed (searched until August 5, 2020). Doses administered to our DC study cohort, and data from healthy individuals (HV) were also used in the development of the dose profile. All these data were incorporated in the predictive regression model for identifying a tentative effective dose range of thiamine (Figure 2).

**FIGURE 3:**
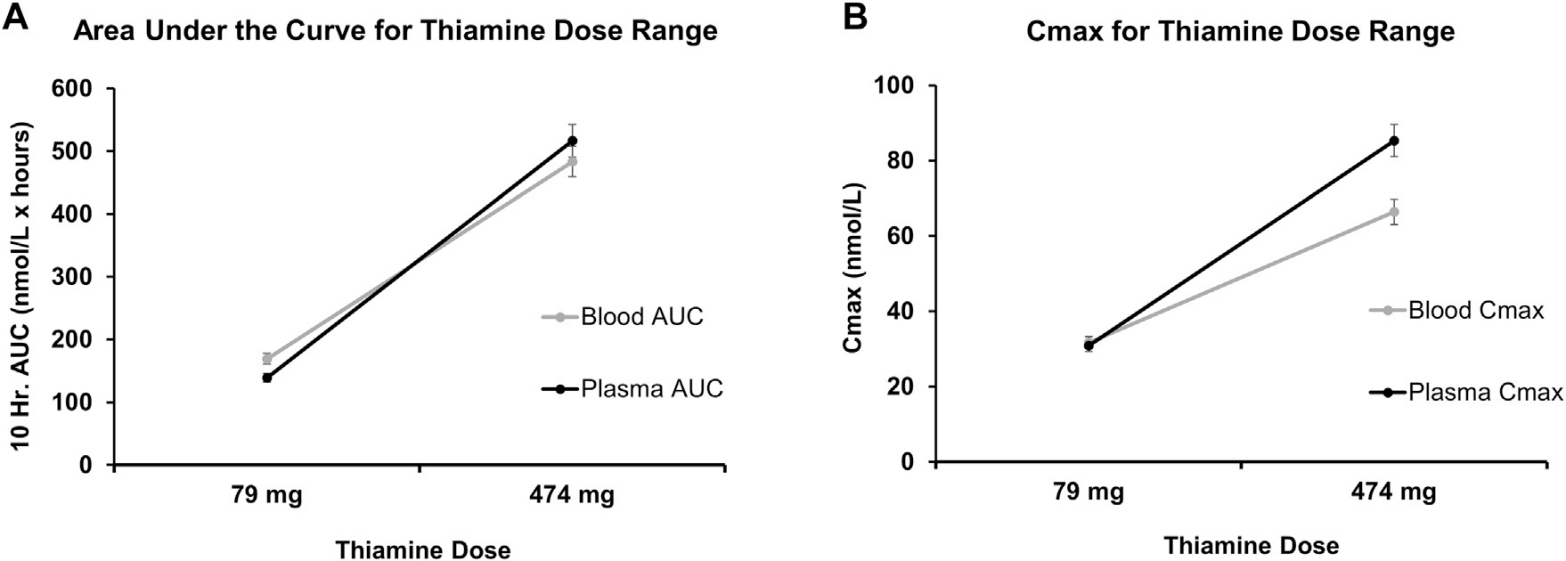
Pharmacokinetics parameters of oral thiamine over 10 h (Projected) in whole blood and plasma. **(A)** Predicted course of Area Under the Curve (AUC) for low values (79 mg/day) and high values (474 mg/day) of the range of oral thiamine dose in blood and plasma over the 10 h. **(B)** Predicted maximum concentration of low values (79 mg/day) and high values (474 mg/day) of the range of oral thiamine dose in blood and plasma over the first 10 h. Errors bars show a 5% variability in the pharmacokinetic measures at each dose.

The pharmacokinetic response of thiamine was calculated at both the low and high ends of the dose range described above. The area under the curve and maximum concentration (*C*_max_) were established for both blood and plasma for a 10-h trajectory (Figure 3) using the indices of thiamine’s *in vivo* blood pharmacokinetics (Smithline et al., 2012). For the derived 79 mg thiamine dosing (low end), the slopes used to identify AUC in blood were 2.14, and 1.76 in plasma. For the 474 mg thiamine dosing (upper end), the slope used to derive AUC in blood was 1.02, while in plasma it was 1.09. Similarly, for the 79 mg thiamine dosing, the slope used to derive *C*_max_ in blood was 0.40, and in plasma it was 0.39. For the 474 mg thiamine dose, the slope used to derive AUC in blood was 0.14, and in plasma it was 0.18.

### Statistical Analysis

Data are expressed as Mean ± standard deviation (M ± SD) in Supplementary Table S1 as well as in Figures 1, and Supplementary Figure S1. Two-sided Student’s t-test was used to examine the difference between disease controls and healthy volunteers at baseline (see Figure 1), and two-sided paired *t*-test was used to examine the changes at baseline vs. 3 weeks for disease controls (Figure 3).

*Post-hoc* one-sided t-tests were performed for the IL-17 and IL-22 mRNA expression analyses for the RAW264.7 cells testing (Supplementary Figure S1). A pharmacokinetic model for anticipated therapeutic dosing range of thiamine based on IL-17 ranges in different inflammatory conditions was constructed using predictive regression computation (Figures 2). Factorial between-group ANOVA was used to evaluate demographic and cytokine profiles (Supplementary Table S1). Statistical significance was established at *p <* 0.05. SPSS 26.0 (IBM Chicago, IL) and Microsoft Excel 365 (MS Corp, Redmond WA), statistical software R (https://www.r-project.org/), and Prism GraphPad (GraphPad Software, San Diego, CA) were used for statistical analysis, data computation, and plotting the figures.

### Neurological Assessments

We conducted a review on the neurological presentation in COVID-19 and other relevant viral occurrences of encephalitis (Table 1). We identified and tabulated the neurological symptoms of COVID-19 and viral encephalitis from the recently published findings. We also described the neurological symptoms, that are generally treated effectively with thiamine (Table 1). We used PubMed and Medline Plus for disease references (searched until August 5, 2020).

**TABLE 1:** Neurological symptoms in COVID-19, Viral Encephalitis (Grouped/assorted by the neurological spectrum observed in COVID-19), showing their proximity in presentation.

Neurological symptoms			Corresponding clinical Indications
COVID-19	Viral encephalitis	Therapeutic effects of thiamine on WKS and other neurological conditions	
Altered mental status			
(Mao et al., 2020; Wang et al., 2020)	Disorientation (Chaudhuri and Kennedy, 2002)	Mental Confusion (Reuler et al., 1985; Sechi and Serra, 2007), impaired Memory (Sechi and Serra, 2007)	Confusion: WKS classic triad
Epiphora, conjunctival congestion, or chemosis (swollen conjunctiva) (Wu et al., 2020); ophthalmoplegia(Lantos et al., 2020) (part of MFS)	Ocular Paralysis (Keane, 2007), internuclear Ophthalmoplegia (Hedges III, 1994; Keane, 2005; Sanjay et al., 2009)	Ophthalmoplegia (nystagmus) (Sechi and Serra, 2007)	Ocular: WKS classic triad
Ataxia (Yeh et al., 2004; Mao et al., 2020) (part of MFS): movement (Lantos et al., 2020), and unstable walking (Wang et al., 2020)	Ataxia (Brechtelsbauer et al., 1997; Chaudhuri and Kennedy, 2002)	Gait Ataxia (Sechi and Serra, 2007)	Ataxia: WKS classic triad
Fatigue (Wang et al., 2020), dizziness and languidness (Mao et al., 2020; Wang et al., 2020), malaise(Wang et al., 2020), headache (Mao et al., 2020; Wang et al., 2020)	Weakness and Somnolence (Brechtelsbauer et al., 1997), Nausea (Whitley and Gnann, 2002)	Lack of energy/Fatigue, drowsiness, fainting, Sluggishness (Sechi and Serra, 2007); Apathy (Sechi and Serra, 2007)	Generalized features
Cerebral hemorrhage (Wang et al., 2020), cerebral infarction (Wang et al., 2020)	Cerebral Hemorrhage (Kabakus et al., 2005), intracranial Pressure (Kumar et al., 2009)	Hemorrhages (Sechi and Serra, 2007)	Cerebrovascular
Epilepsy (Hao et al., 2020; Mao et al., 2020)	Epilepsy (Aguilar and Rasmussen, 1960; Misra et al., 2008)	Seizures (Alcohol Withdrawal) (Nguyen and Lam, 2020)	Pathophysiological
Encephalomyelitis: demyelinating (Steardo et al., 2020; Wang et al., 2020), disseminated (Reichard et al., 2020)	Encephalomyelitis (Whitley, 1990; Dale, 2003)	Demyelination (Rao and Topiwala, 2020) within periventricular structures	Pathomorphological
Hypogeusia (low ability to taste)			
(Finsterer and Stollberger, 2020; Gautier and Ravussin, 2020; Lechien et al., 2020; Mao et al., 2020)	Hypogeusia (low ability to taste) (Henkin et al., 1975)	Efficacy not well-established	Sensory
Hyposmia (low ability to smell) (Bénézit et al., 2020; Finsterer and Stollberger, 2020; Lechien et al., 2020; Mao et al., 2020)	Hyposmia (low ability to smell) (Henkin et al., 1975)	Efficacy not well-established	Sensory
Nerve pain (Azhideh, 2020) (also in the head and face region (Mao et al., 2020))	Neuralgia (Johnson et al., 2010; Tang et al., 2013)	Neuralgia (Rose and Jacobson, 1940; Eckert and Schejbal, 1992)	Neuralgia
Tachycardia (He et al., 2020; Kochi et al., 2020)	Tachycardia (Chua et al., 1999; Chaudhuri and Kennedy, 2002)	Racing of heart (faster heartbeat), low blood pressure (Sechi and Serra, 2007), Tachycardia (Sechi and Serra, 2007)	Cardiovascular
Muscle injury			
(Cascella et al., 2020; Mao et al., 2020)	Dysarthria (Brechtelsbauer et al., 1997), nerve Impairment (Ripamonti et al., 2020)	Motor impairment (Pitel et al., 2011), motor cortex Excitability (Nardone et al., 2010)	Motor
Areflexia (part of MFS) (Lantos et al., 2020)			

Therapeutic effects of thiamine on the corresponding neurological symptoms of pro-inflammatory origin that are also observed in viral infection.

WKS: Wernicke Korsakoff Syndrome (Blass and Gibson, 1977); MFS: Miller Fisher Syndrome (Mori et al., 2001).

## Results

### Demographics and Candidate Proinflammatory Cytokine Profile

DC group individuals in this study had significantly higher age than the healthy controls (HV) (Supplementary Table S1). However, there was no significant difference in the mean BMIs between the two groups, and the sex-distribution was also similar between the two groups. DC group individuals drank 1,096.58 ± 505.71 drinks in the past 90 days (around 12 drinks daily). Both IL-6 (∼6 fold) and IL-1β (∼3 fold) cytokines were significantly higher in the DC group (AUD patients) compared to the healthy controls/volunteers (HV) group. IL-10 was also numerically higher in the DC group.

### Clinical Findings on the Immune Response of Th17 Derived IL-17 and IL-22 Axis Response, and Thiamine Efficacy and Safety

To develop a model for thiamine effects on inflammation, we assessed IL-17 and IL-22 cytokine expression (showing proinflammatory and anti-inflammatory effects, respectively). Both cytokines are produced by the Th17 cells (Qu et al., 2013). IL-17 concentrations were below the level of detection in the HV group but were elevated in the DC group (Figure 1A). An approximate four-fold decrease was observed in the IL-17 concentration levels (0.09 pg/ml to 0.023 pg/ml) with a treatment dose of 200 mg thiamine daily (Estimated AUC = 204 nmol/L x hour approximately in the 10-h window) by the end of week 3. IL-22 was significantly decreased in DC group compared to healthy volunteers and thiamine therapy did not significantly improve levels in treated DC group individuals (Figure 1B). No patients reported any kind of drug related adverse events; therefore, the safety profile of thiamine administration was excellent at 200 mg daily in this small pilot group.

### IL-17 Dependent Dose and Pharmacokinetic Model of Thiamine

An IL-17 response dependent dose and pharmacokinetic model of thiamine administration was developed, based on responses from the pro-inflammatory disease cohorts and the corresponding thiamine dosing (controlled for by the corresponding values in healthy volunteers, as a point of reference). This model supported a tentative range of thiamine dosing for COVID-19 (Figures 2A,B), since the IL-17 levels are much higher in COVID-19 than in the reports from many other proinflammatory disease conditions. Using regression analysis, a range of 79 mg/day (lower end of dose range)−474 mg/day (higher end of dose range) for thiamine administration was found to correspond to a range of 15–40 ng/ml level of IL-17 used *in vitro*.

The pharmacokinetic parameters were: Area Under the Curve (AUC) (Figure 3A), and Maximum (or peak) Concentration of a drug (C_max_) (Figure 3B). These gave a very close estimation of the specific oral thiamine dose (Figure 2). As expected, plasma values were higher for both AUC and C_max_ at higher doses and were lower at lower doses (Supplementary Table S2).

### Assessment of Neurological Presentation of COVID-19, Viral Encephalitis and Therapeutic Efficacy of Thiamine Administration

We tabulated the neurological symptoms from the recently published findings on COVID-19 (Table 1). Severely ill COVID-19 patients presented with various neurologic symptoms that could be grouped together as: acute cerebrovascular disease; altered mental status; and musculoskeletal symptoms (Mao et al., 2020). We also included the neurological presentation commonly observed in viral encephalitis of non-COVID-19 origin, grouped corresponding to the presentation of the symptoms of COVID-19.

Lastly, we also tabulated the neurological symptoms that are commonly treated with thiamine. Several neurological symptoms of COVID-19 and viral encephalitis corresponded well with the neurological spectrum that is known to be managed effectively with thiamine.

## Discussion

We evaluated individuals with significantly altered IL-17 and IL-22 responses associated with Th17 cells and found a significant role for a 3-weeks 200 mg/daily thiamine treatment regimen in improving the Th17 response in the AUD disease control patient cohort whose members exhibited a high pro-inflammatory status at baseline. SARS CoV-2 viral challenge causes induction of IL-6 leading to altered Th17 responses (Hotez et al., 2020). IL-17 synthesized by Th17 cells can markedly stimulate neutrophil chemotaxis and may lead to a skewed Th2 immune response (Song and Qian, 2013; Veldhoen, 2017). Results from our AUD control group show the increases in candidate proinflammatory (IL-1β and IL-17), and anti-inflammatory (IL-6, IL-10, and IL-22) cytokines. Changes in IL-17 and IL-22 with alcohol abuse (pro-inflammatory), and thiamine as an anti-inflammatory therapy in our experiment provided potential proof of concept. IL-1β is a key cytokine initiating Th17 cells to synthesize IL-17 (Lasiglie et al., 2011), whereas IL-10 suppresses Th17 proinflammatory cytokine production (Gu et al., 2008). High IL-6 levels also are associated with Th17 cell proinflammatory activity (Hotez et al., 2020). Thus, under inflammatory conditions, there is a complex interaction of proinflammatory/anti-inflammatory responses from Th17 cells. An intervention or prevention that could attenuate the Th17 proinflammatory activity could help ameliorate the consequences of a cytokine storm.

Thiamine deficiency has been reported to promote a proinflammatory response in Th1 and Th17 cells (Ji et al., 2014). To examine the role of thiamine in treating inflammation, *in vitro* testing was used to mechanistically examine the clinical outcomes. Our *invitro* model hinted that thiamine could lower IL-17 and increase IL-22 mRNA expression in macrophages. Our clinical data suggest that thiamine could play a potential role in attenuating the cytokine storm in patients who have a strong proinflammatory response.

A study using a mouse model showed that IL-17 augments respiratory syncytial virus (RSV)-induced lung inflammation (Mebratu and Tesfaigzi, 2018). In that study, immunodepletion of IL-17 before viral infection resulted in diminished RSV-driven mucous cell hyperplasia and airspace enlargement, suggesting IL-17 as a potential therapeutic target. Proinflammatory IL-17 production could also initiate pulmonary eosinophilic response, by promoting proliferation of eosinophils in the bone marrow, followed by recruitment and extravasation into the lungs (Murdock et al., 2012). In MERS-CoV, SARS-CoV and SARS-CoV-2, disease severity showed positive correlations with the levels of IL-17 and other T helper 17 (Th17) cell-related pro-inflammatory cytokines, such as IL-1, IL-6, IL-15, TNF and IFNγ (Mahallawi et al., 2018; Liu et al., 2020b). Dysregulated Th17 cells and IL-17 synthesis in the skin, synovial space and endothelium promote synthesis of pro-inflammatory cytokines namely IL-1β, TNF and IL-6 and neutrophil chemoattractants such as IL-8, CCL20 and CCL2 as observed in psoriasis (Gaspari and Tyring, 2015; Silfvast-Kaiser et al., 2019) and psoriatic arthritis (Blauvelt and Chiricozzi, 2018; Pacha et al., 2020). A similar response is also observed in ARDS, with IL-17 involvement of lung parenchyma damage, stimulatory synthesis of the proinflammatory mediators, and by the inhibition of apoptosis due to the enhanced expression of the colony-stimulating factor (Muir et al., 2016). IL-17 related viral myocarditis (Guzik et al., 2020) is also exhibited in COVID-19 (Zeng et al., 2020). A dominant Th17 phenotype response could drive more severe viral myocarditis (Myers et al., 2016) thus leading to the multi-organ effects of Th17 proinflammatory response (Siripanthong et al., 2020). These recent findings suggest that suppression of IL-17 may be vital to managing viral infections, including COVID-19 and their harmful consequences. Thus, targeting IL-17 immunologically can be an effective strategy to prevent acute respiratory distress syndrome (ARDS) in coronavirus disease 2019 (COVID-19) (Pacha et al., 2020). There are treatment for IL-17 such as IL17 inhibitors (Hohenberger et al., 2018) that are used as standard of care for certain inflammatory conditions (Ly et al., 2019); and further drug repurposing investigation are underway (Hohenberger et al., 2018) including for the viral infection (de Almeida Nagata et al., 2014) and COVID-19 (Bulat et al., 2020). However, there are some limitations as adverse events that limit its scope for SOC and repurposing such as likelihood of nasopharyngitis, infections (Loft et al., 2020), major adverse cardiovascular events (MACE) (Zeng et al., 2020), inflammatory bowel disease (IBD) are some of the prominent AEs (Loft et al., 2020).

We derived an effective dose range of thiamine that could be administered for alleviating the Th17 cell proinflammatory response by using the IL-17 concentrations that we obtained from our AUD patients (termed as DC), levels found in the literature, and levels in the healthy control (HV) group. Thiamine has been administered as a treatment in other viral infections (Butterworth et al., 1991; Shoji et al., 1994), and has proven effective for some inflammatory conditions and symptoms (Wallace and Weeks, 2001; Kim et al., 2018). A well-structured treatment profile of thiamine based on the results of proof of concept *in vitro* experiments, and analyses of proinflammatory response-relevant disease conditions support the potential efficacy of thiamine in ameliorating the proinflammatory Th17 response in severe COVID-19 patients. Use of preventive as well as interventional dosages show potential in the management of COVID-19. Thiamine C_trough_ is reached generally in 10–12 h; thus, the total dose prescribed can be divided into two doses per day. This may lead to fewer AEs or other side effects. Thiamine has now been included in the standard of care protocol for treating cytokine storm in severe COVID-19 patients (Group, 2020). Unlike the IL-17 inhibitors as mentioned above, Thiamine has no side effects. It also can be formulated easily in high dose and delivery has convenient oral and IV routes.

One recent report identified that 36.4% of the COVID-19 diagnosed patients have neurologic symptoms, and this proportion was higher (45.5%) among those COVID-19 patients with more severe symptoms (Mao et al., 2020). Patients with other viral diseases have also shown clinical symptoms of beriberi (Coates et al., 2010), or Wernicke–Korsakoff syndrome (autopsies of 380 people with AIDS showed Wernicke’s encephalopathy in 10% of the cases) (Boldorini et al., 1992), and these conditions are associated with thiamine deficiency. It is possible that patients with viral infection could have an increased risk of thiamine deficiency, but this information has remained largely unexplained in viral diseases (Larsen et al., 2013), including COVID-19. A potential reason could be that a deficiency in thiamine could be related to the thiamine transport protein, which can have a general preference for multiple membrane transport molecules which can function as receptors for candidate viruses (Mendoza et al., 2006). The Th17 proinflammatory response has also been reported in the experimental encephalomyelitis model (Ji et al., 2014). Thus, thiamine could be a therapeutic agent to alleviate neurological symptoms of COVID-19.

Adverse effects (AE) of thiamine are minimal and generally mild. Possible AEs include nausea, diarrhea, and abdominal pain. Rarely, individuals also suffer serious allergic reactions. There are no reported drug related symptoms at the 200 mg thiamine dose used in our study, and there are no reported AEs. A landmark pharmacokinetic study utilizing a 1,500 mg maximum oral dose of thiamine in healthy subjects showed rapid absorption (Smithline et al., 2012). Moreover, 4,000 mg thiamine administration showed no to mild AEs when used in children (Leigh’s disease). Thus, higher doses of thiamine for treatment of COVID-19’s cytokine storm could be considered a safe therapy.

Our study has several limitations. This is a small study; thus, the outcomes have limited analytical and derivation scope. However, both clinical and *in vitro* evidence collectively support the potential of thiamine as a therapeutic agent in attenuating the Th17 proinflammatory response. We did not test the *invitro/in vivo* efficacy of Thiamine in the treatment of COVID-19 or its derivative stimulated Th17 proinflammatory response directly. We anticipate conducting such *invitro* experiments for COVID-19 as a continuation of this project, where we will test the effective dose in the *exvivo* and *invitro* model. Moreover, plasma thiamine levels have not been assessed in COVID-19. The healthy volunteer group was mostly younger and did not have older individuals. Our study did not have sufficiently large numbers of males and females; thus, identifying sex-differences was not within the scope of this study. This is a proof of concept study on one therapeutic agent, thus results from COVID-19 patients on the involvement of immune response is not in the scope of this study. We intend to conduct the pre-clinical investigation with the specimens from the COVID-19 patients; and experimental design involving COVID-19 treated cell response for the IL-17 expression changes, respectively.

In summary, Thiamine has been approved by the Food and Drug Administration (FDA) of the USA as a prescription product and is considered very safe even at higher doses since it is water soluble and can be excreted via urine, if in excess (Plaut, 1961). Given its robust safety record, we suggest that thiamine should be considered for COVID-19 treatment studies.

## Data Availability

The raw data supporting the conclusions of this article will be made available by the authors, without undue reservation.
