# Supplementary material for: Repurposing Treatment of Wernicke–Korsakoff Syndrome for Th-17 Cell Immune Storm Syndrome and Neurological Symptoms in COVID-19: Thiamine Efficacy and Safety, In-Vitro Evidence and Pharmacokinetic Profile
Source: Front Pharmacol. 2021 Mar 2;11:598128. doi: 10.3389/fphar.2020.598128 (PMC7960760; doi:10.3389/fphar.2020.598128)
Supplement: Supplementary file 1 [file datasheet1.pdf]

Supplementary Section

Results

Experimental Model for Treatment Efficacy of Thiamine on IL-17 and IL-22 Activity

Using these clinical findings of the treatment efficacy of thiamine, we designed an *invitro* experiment using mouse macrophage RAW264.7 cells to validate the effects of thiamine on alcohol-induced IL-17 and IL-22 expression. The results showed that a low dose of thiamine (V_{B1}) decreased IL-17 expression in the absence (by 20% approximately) and/or presence of alcohol treatment (by 25% roughly) (Supple Figure 1a). Importantly, IL-22 expression was upregulated by thiamine in both the control and ethanol-treated cells. This response in IL-22 expression was moderately reduced by alcohol but rescued by the thiamine (V_{B1}) treatment (Supple. Figure 1b). Half of the treatment dose (0.02 µg/mL) that was found to be effective in humans was beneficial as a preventative dose (0.01 µg/mL) of thiamine in this *in vitro* model.

Suppl Table 1. Presentation of demographic and candidate proinflammatory cytokines in the study participants.

Measures	Disease Controls (n=16)	Healthy Controls (n=8)	Comments
Age (yrs.)	41.87±11.04	28.38±4.44	0.002
Sex (F/M), in number	F=4/M=12	F=2/M=6	NA
BMI (units)	27.11±6.14	24.65±3.328	NS
Candidate Cytokines observed in immune storm			
IL-6 (pg/ml)	2.478±1.37	0.406±0.20	≤0.001
IL-10 (pg/ml)	0.335±0.17	0.285±0.12	NS
IL-1β (pg/ml)	0.598±0.30	0.219±0.40	0.017

Footnote - yrs.: years; F: Females; M: Males; BMI: Body Mass index; IL: Interleukin; β: beta; pg: picogram; ml: milliliter; NA: Not Applicable; NS: Not significant.

Suppl Table 2. Area Under the Curve (AUC), and maximum or peak concentration (C_{max}) derived for the low and high doses of thiamine (Vit B1).

Vit B1 Dose	79 mg	474 mg
Blood AUC<br><br>Plasma AUC	169.06<br><br>139.04	483.48<br><br>516.66
CL	56%	91%
Blood C_{max}	31.6	66.36
Plasma C_{max}	30.81	85.32

Footnote - Unit for AUC: nmol/Liter x Hours for 10 Hrs.; CL: Clearance; Unit for C_{max}: nmol/Liter; AUC: Area Under the Curve; C_{max}: Peak Concentration of Drug in Specific Body System/ Organ. Vit B1: Thiamine; mg: milligram.

Supplement Figure 1 legend: mRNA expression of IL-17 and IL-22 in an *invitro* model of the mouse macrophage cell line using a potent proinflammatory agent (alcohol), and preventative administration of thiamine. SFig. 1a: Significant lowering was observed in IL-17 mRNA

expression in “EtOH + V_{B1}” (Bar 4) group compared to the alcohol-treated group (Bar 3). SFig. 1b: Significant elevation was observed in IL-22 mRNA expression in “EtOH + V_{B1}” (Bar 4) group compared to the alcohol-treated group (Bar 3). Controls (both SFig. 1a and 1b) show normal anti-inflammatory effects of thiamine on IL-17 and IL-22 response with thiamine administration. Ctrl: Non-treated controls. V_{B1} 0.01 ug/ml. EtOH: alcohol treated. V_{B1}+E: thiamine and alcohol treated. Data are presented as M±SD. Statistical significance was set as $p < 0.05$.
