# Supplementary figures and images for: Repurposing Treatment of Wernicke–Korsakoff Syndrome for Th-17 Cell Immune Storm Syndrome and Neurological Symptoms in COVID-19: Thiamine Efficacy and Safety, In-Vitro Evidence and Pharmacokinetic Profile
Source: Front Pharmacol. 2021 Mar 2;11:598128. doi: 10.3389/fphar.2020.598128 (PMC7960760; doi:10.3389/fphar.2020.598128)

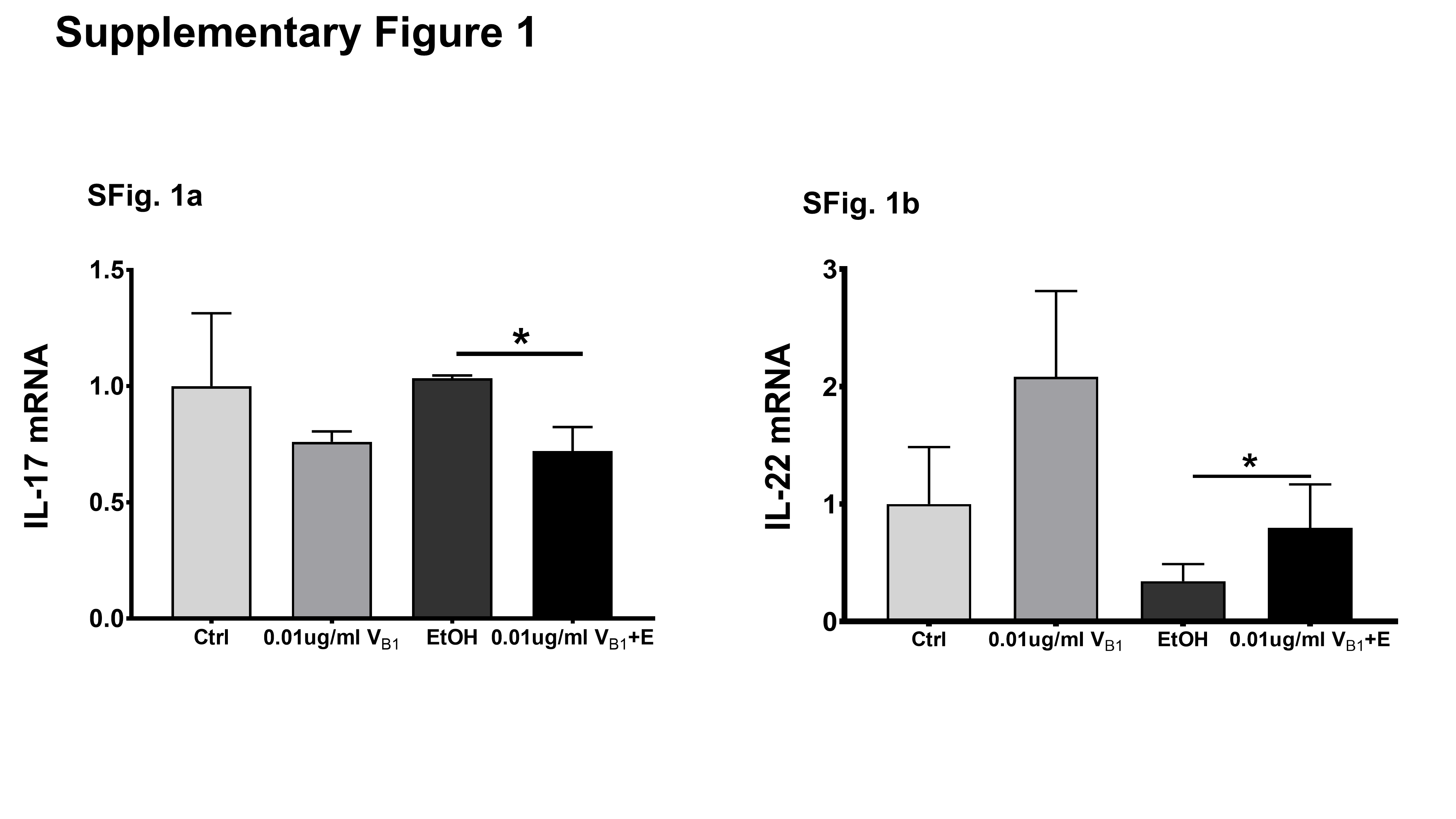

Supplement: Supplementary file 2 [file image1.tif]
